# Synthetic biology beyond borders

**DOI:** 10.1111/1751-7915.13966

**Published:** 2021-11-18

**Authors:** Karthik Raman, Himanshu Sinha, Claudia E. Vickers, Pablo I. Nikel

**Affiliations:** ^1^ Department of Biotechnology Centre for Integrative Biology and Systems Medicine (IBSE) Indian Institute of Technology Madras Chennai India; ^2^ CSIRO Future Science Platform in Synthetic Biology Commonwealth Scientific and Industrial Research Organization (CSIRO) Dutton Park Australia; ^3^ The Novo Nordisk Foundation Center for Biosustainability Technical University of Denmark Kongens Lyngby Denmark

Underlying every living cell, there is a beautifully concerted circuitry of signalling, metabolism and regulation that, together, orchestrate a variety of functions. Synthetic biology has targeted this circuitry at all levels, engineering it towards a wide array of applications – but also expanding our understanding fundamental aspects of the cell. Fuelled by advances in DNA sequencing, synthesis, editing, and computational modelling and circuit design, synthetic biology has broken tremendous ground in the last decade all across the world.

The same way engineers attempt to understand and create complex systems, biologists have strived to dissect and understand the complexities of biological systems. Bringing together both engineers and biologists with diverse backgrounds, the highly successful India‐EMBO symposium ‘Engineering meets evolution: Designing biological systems’ was hosted at Chennai, India, in January 2020 – one of the last opportunities to gather in a physical meeting just before the COVID pandemic broke loose. Actually, the rapid and efficient rollout of vaccines designed to handle the COVID crisis is a very palpable example of how synthetic biology can have a positive impact on our daily lives (Brüssow, 2021). Born out of the exciting discussions during the symposium, ‘*Synthetic biology beyond borders*’, the current issue of *Microbial Biotechnology*, collates a number of interesting advances in the field from contributors all around the world. Composed by both review and research articles, this special issue covers topics ranging from efficient genome editing of cell factories to metabolic engineering for the production of complex, value‐added molecules – with authors from 15 different countries in Europe, America and Asia. As synthetic biology continues to expand horizons, the importance of a special issue that covers a breadth of topics in the field cannot be overstated.

## Synthetic biology: moving biology from phenomenology towards engineering

Ever since the first gene circuits were designed and constructed (Elowitz and Leibler, 2000; Gardner and Cantor, 2000), synthetic biology brought about an engineering approach to biology (Danchin, 2021) – whereby sophisticated behaviours can be designed into biological systems (de Lorenzo and Danchin, 2008; Stephanopoulos, 2012; Kahl and Endy, 2013; Church *et al*., 2014; Flores Bueso and Tangney, 2017; Huang and Nikel, 2019; Wurtzel *et al*., 2019). A wide range of switches, oscillators, logic gates, filters and even pulse generators have been incorporated into living cells under this conceptual framework. The defining aspect of synthetic biology is the rational design of biological circuitry using existing or novel genes, in a predictive fashion, with the ultimate goal of making biology more and more *engineerable* – where various standardized ‘parts’ and ‘modules’ can be reliably composed, physically and functionally, to yield ‘devices’ that display desired functionalities (Arkin, 2008; Silva‐Rocha *et al*., 2013; Decoene *et al*., 2018). In this regard, synthetic biologists have to deal with an unavoidable aspect of living systems, that is, their capacity to evolve (Nørholm, 2019; Sambamoorthy and Sinha, 2019). As illustrated in a research article in this special issue, adaptive laboratory evolution is, on itself, a powerful tool for the design and optimization of cell factories. Evolution is an inherent property of every cell platform used for synthetic biology, and the plethora of *chassis* currently available supported the expansion of the range of practical applications (Danchin, 2012; Calero and Nikel, 2019; Abram and Udaondo, 2020). A research article in this special issue describes the genome and phenotypic characterization of yet another bacterial platform, *Pseudomonas umsongensis* GO16, a metabolically versatile *Pseudomonas* species. Synthetic microbial communities are likewise gaining increasing attention (Ibrahim and Raajaraam, 2021; Kumar *et al*., 2021). These elements serve as the basis for the *Design–Build–Test–Learn* (DBTL) cycle that has become the cornerstone in the field, especially in the context of biofoundries (Holowko *et al*., 2021) and some well‐funded companies that are currently driving automation of synthetic biology. All these efforts are matched with the emerging role of artificial intelligence and deep learning – expected to make a major impact in the DBTL cycle, especially at the *Design* phase.

## The multiple fundamental and applied facets of synthetic biology

When synthetic biology emerged as an independent scientific field, scientists devoted quite some effort in developing tools for engineering living systems. This special issue includes articles that describe elegant examples of this sort, for example, synthetic promoters that respond to the availability of phosphorus, novel standard vectors for the efficient production of disulphide‐containing proteins and CRISPR (clustered regularly interspaced short palindromic repeats) interference (CRISPRi) in *Pseudomonas*, a transposon toolbox supported by Cre/Lox recombination and counter‐selection towards genome reduction, synthetic modulators of bacterial gene expression, and CRISPR/Cas9 and Cas12a‐assisted protocols for genome engineering in filamentous fungi and photosynthetic bacteria. Computational synthetic biology has been an important cog in developing the field rapidly (Marchisio and Stelling, 2009) – making use of the rich toolbox indicated above. Iterative DBTL cycles, rooted in computational modelling, have enabled the construction of many a circuit, increasing the complexity and diversity of the gene circuitry architecture. Going forward, it will be vital to construct better genome‐scale reconstructions and gene expression models, which could faithfully capture key interactions and cross‐talk within the cell in order to more accurately reflect the performance of designer modules.

These endeavours require a deeper understanding of the general cell processes underlying microbial phenotypes. Such is the case of the 5′‐regulatory regions present in DNA sequences (acting at the transcriptional level of regulation), ribonucleases (involved in post‐transcriptional regulation) and catabolic repression (exerting effects at the metabolic, global level of regulation) – in addition to the redundancy in metabolic activities within central carbon metabolism. Four articles in our special issue discuss these aspects in the light of engineering regulatory traits in microbes.

Perhaps, the most industrially relevant application of synthetic biology lies in the development of microbes capable of synthesizing a variety of molecules, for example, drugs. A classic example is that of S*accharomyces cerevisiae* engineered to produce artemisinic acid, the precursor of a key antimalarial drug (Ro *et al*., 2006). The number and type of chemicals that can be produced with synthetic biology continue to expand (Prather, 2019), impacting not only research but also commercialization (Carbonell *et al*., 2016; Martinelli and Nikel, 2019). In this issue, we have a number of research articles covering synthetic biology‐guided metabolic engineering applications in a variety of microorganisms and target products, that is, *S. cerevisiae* (tyrosol, salidroside, vindoline and *cis*,*cis*‐muconic acid), *Yarrowia lipolytica* (sesquiterpene, bisabolene and aromatic amino acids), *Pseudomonas putida* (5‐ketofructose and vanillylamine), *Mycolicibacterium smegmatis* (steroids), *Streptomyces albus* (salinomycin), *Saccharopolyspora pogona* (butenyl‐spinosyn), *Shewanella oneidensis* (Pd/Ag and Pd/Au nanoparticles) and *Eubacterium limosum* (butyrate). These studies exploit a wide range of strategies to achieve improved titres and illustrate the utility of deploying toolboxes specifically tailored for different *chassis*. In addition, the state of the art in the metabolic engineering of amino acid and small peptide production is covered in two separate review articles. Programing of biosensors for accelerating cell factory optimization, sensing DNA damage and for general applications in environment, health and biomanufacturing is likewise discussed in three articles of this volume.

## Outlook: widespread adoption of synthetic biology beyond borders

While the benefits of synthetic biology outweigh the potential pitfalls, there are a number of key challenges that need to be surmounted in this decade, as synthetic biology goes more mainstream. The actual impact that scientific advances can have on the society involves social and political aspects in connection to adopting new technologies and how to use them in the context of responsible research and innovation (Gregorowius and Deplazes‐Zemp, 2016). The overall perception of synthetic biology and its acceptance by consumers will be key to this end (Gallup and Ming, 2021). There are also regulatory challenges and safety concerns, which need to be dispelled by engaging scientists with the public and policymakers in a two‐way dialogue and through proper education and diffusion of emerging technologies. We hope that such efforts will be shared by the global synthetic biology community beyond territorial and conceptual borders, especially when synthetic biology is scaled‐up to offer global solutions (de Lorenzo and Marlière, 2016). Meanwhile, the examples collected in this special issue illustrate the current status of a burgeoning field that will continue to expand in the years to come and *Microbial Biotechnology* will continue to serve as a dynamic platform to communicate and discuss these developments.

## Conflict of interest

None declared.

## References

[mbt213966-bib-0001] Abram, K.Z. , and Udaondo, Z. (2020) Towards a better metabolic engineering reference: the microbial *chassis* . Microb Biotechnol 13: 17–18. 30589218 10.1111/1751-7915.13363 PMC6922515

[mbt213966-bib-0002] Arkin, A. (2008) Setting the standard in synthetic biology. Nat Biotechnol 26: 771–774. 18612298 10.1038/nbt0708-771

[mbt213966-bib-0003] Brüssow, H. (2021) What we can learn from the dynamics of the 1889 'Russian flu' pandemic for the future trajectory of COVID‐19. Microb Biotechnol. In press. 10.1111/1751-7915.13916 PMC8601188 34464023

[mbt213966-bib-0004] Calero, P. , and Nikel, P.I. (2019) Chasing bacterial *chassis* for metabolic engineering: a perspective review from classical to non‐traditional microorganisms. Microb Biotechnol 12: 98–124. 29926529 10.1111/1751-7915.13292 PMC6302729

[mbt213966-bib-0005] Carbonell, P. , Gök, A. , Shapira, P. , and Faulon, J.L. (2016) Mapping the patent landscape of synthetic biology for fine chemical production pathways. Microb Biotechnol 9: 687–695. 27489206 10.1111/1751-7915.12401 PMC4993189

[mbt213966-bib-0006] Church, G.M. , Elowitz, M.B. , Smolke, C.D. , Voigt, C.A. , and Weiss, R. (2014) Realizing the potential of synthetic biology. Nat Rev Mol Cell Biol 15: 289–294. 24622617 10.1038/nrm3767 PMC9645560

[mbt213966-bib-0007] Danchin, A. (2012) Scaling up synthetic biology: do not forget the *chassis* . FEBS Lett 586: 2129–2137. 22226636 10.1016/j.febslet.2011.12.024

[mbt213966-bib-0008] Danchin, A. (2021) *In vivo*, *in vitro* and *in silico*: an open space for the development of microbe‐based applications of synthetic biology. Microb Biotechnol. In press. 10.1111/1751-7915.13937 PMC8719824 34570957

[mbt213966-bib-0009] Decoene, T. , De Paepe, B. , Maertens, J. , Coussement, P. , Peters, G. , De Maeseneire, S.L. , and De Mey, M. (2018) Standardization in synthetic biology: an engineering discipline coming of age. Crit Rev Biotechnol 38: 647–656. 28954542 10.1080/07388551.2017.1380600

[mbt213966-bib-0010] Elowitz, M.B. , and Leibler, S. (2000) A synthetic oscillatory network of transcriptional regulators. Nature 403: 335–338. 10659856 10.1038/35002125

[mbt213966-bib-0011] Flores Bueso, Y. , and Tangney, M. (2017) Synthetic biology in the driving seat of the bioeconomy. Trends Biotechnol 35: 373–378. 28249675 10.1016/j.tibtech.2017.02.002

[mbt213966-bib-0012] Gallup, O. , Ming, H. , and Ellis, T. (2021) Ten future challenges for synthetic biology. Eng Biol 5: 51–59. 10.1049/enb2.12011 PMC9996719 36968258

[mbt213966-bib-0013] Gardner, T.S. , Cantor, C.R. , and Collins, J.J. (2000) Construction of a genetic toggle switch in *Escherichia coli* . Nature 403: 339–342. 10659857 10.1038/35002131

[mbt213966-bib-0014] Gregorowius, D. , and Deplazes‐Zemp, A. (2016) Societal impact of synthetic biology: responsible research and innovation (RRI). Essays Biochem 60: 371–379. 27903824 10.1042/EBC20160039

[mbt213966-bib-0015] Holowko, M.B. , Frow, E.K. , Reid, J.C. , Rourke, M. , and Vickers, C.E. (2021) Building a biofoundry. Synth Biol 6: ysaa026. 10.1093/synbio/ysaa026 PMC7998708 33817343

[mbt213966-bib-0016] Huang, W.E. , and Nikel, P.I. (2019) The *Synthetic Microbiology Caucus*: from abstract ideas to turning microbes into cellular machines and back. Microb Biotechnol 12: 5–7. 30461208 10.1111/1751-7915.13337 PMC6302714

[mbt213966-bib-0017] Ibrahim, M. , Raajaraam, L. , and Raman, K. (2021) Modelling microbial communities: harnessing consortia for biotechnological applications. Comput Struct Biotechnol J 19: 3892–3907. 34584635 10.1016/j.csbj.2021.06.048 PMC8441623

[mbt213966-bib-0018] Kahl, L.J. , and Endy, D. (2013) A survey of enabling technologies in synthetic biology. J Biol Eng 7: 13. 23663447 10.1186/1754-1611-7-13 PMC3684516

[mbt213966-bib-0019] Kumar, N. , Hitch, T.C.A. , Haller, D. , Lagkouvardos, I. , and Clavel, T. (2021) MiMiC: a bioinformatic approach for generation of synthetic communities from metagenomes. Microb Biotechnol 14: 1757–1770. 34081399 10.1111/1751-7915.13845 PMC8313253

[mbt213966-bib-0020] de Lorenzo, V. , and Danchin, A. (2008) Synthetic biology: discovering new worlds and new words. EMBO Rep 9: 822–827. 18724274 10.1038/embor.2008.159 PMC2529360

[mbt213966-bib-0021] de Lorenzo, V. , Marlière, P. , and Solé, R. (2016) Bioremediation at a global scale: from the test tube to planet Earth. Microb Biotechnol 9: 618–625. 27489146 10.1111/1751-7915.12399 PMC4993180

[mbt213966-bib-0022] Marchisio, M.A. , and Stelling, J. (2009) Computational design tools for synthetic biology. Curr Opin Biotechnol 20: 479–485. 19758796 10.1016/j.copbio.2009.08.007

[mbt213966-bib-0023] Martinelli, L. , and Nikel, P.I. (2019) Breaking the state‐of‐the‐art in the chemical industry with new‐to‐Nature products *via* synthetic microbiology. Microb Biotechnol 12: 187–190. 30706666 10.1111/1751-7915.13372 PMC6389841

[mbt213966-bib-0024] Nørholm, M.H.H. (2019) *Meta* synthetic biology: controlling the evolution of engineered living systems. Microb Biotechnol 12: 35–37. 30548798 10.1111/1751-7915.13350 PMC6302734

[mbt213966-bib-0025] Prather, K.L.J. (2019) Chemistry as biology by design. Microb Biotechnol 12: 30–31. 30488636 10.1111/1751-7915.13345 PMC6302718

[mbt213966-bib-0026] Ro, D.K. , Paradise, E.M. , Ouellet, M. , Fisher, K.J. , Newman, K.L. , Ndungu, J.M. , *et al*. (2006) Production of the antimalarial drug precursor artemisinic acid in engineered yeast. Nature 440: 940–943. 16612385 10.1038/nature04640

[mbt213966-bib-0027] Sambamoorthy, G. , Sinha, H. , and Raman, K. (2019) Evolutionary design principles in metabolism. Proc Biol Sci 286: 20190098. 30836874 10.1098/rspb.2019.0098 PMC6458322

[mbt213966-bib-0028] Silva‐Rocha, R. , Martínez‐García, E. , Calles, B. , Chavarría, M. , Arce‐Rodríguez, A. , de las Heras, A. , *et al*. (2013) The Standard European Vector Architecture (SEVA): a coherent platform for the analysis and deployment of complex prokaryotic phenotypes. Nucleic Acids Res 41: D666–D675. 23180763 10.1093/nar/gks1119 PMC3531073

[mbt213966-bib-0029] Stephanopoulos, G. (2012) Synthetic biology and metabolic engineering. ACS Synth Biol 1: 514–525. 23656228 10.1021/sb300094q

[mbt213966-bib-0030] Wurtzel, E.T. , Vickers, C.E. , Hanson, A.D. , Millar, A.H. , Cooper, M. , Voss‐Fels, K.P. , *et al*. (2019) Revolutionizing agriculture with synthetic biology. Nat Plants 5: 1207–1210. 31740769 10.1038/s41477-019-0539-0

